# Curcumin prevents strokes in stroke-prone spontaneously hypertensive rats by improving vascular endothelial function

**DOI:** 10.1186/s12872-018-0768-6

**Published:** 2018-03-01

**Authors:** Cong Lan, Xinjian Chen, Yuxun Zhang, Wei Wang, Wei Eric Wang, Yukai Liu, Yue Cai, Hongmei Ren, Shuo Zheng, Lin Zhou, Chunyu Zeng

**Affiliations:** 1 Department of Cardiology, Daping Hospital, Third Military Medical University, 10 Changjiangzhilu Road, Yuzhong District, Chongqing, 400042 China; 2 Chongqing Institute of Cardiology, Chongqing, China; 3 Department of Cardiology, 458 Hospital of People’s Liberation Army, Guangzhou, China; 4 Department of Cardiology, the hospital of PLA N.O.95877, 14 branch road, Jiuquan, Gansu 735018 China

**Keywords:** Endothelial function, Oxidative stress, SHRsp, Stroke, UCP2

## Abstract

**Background:**

Antioxidants have shown great promise in stroke prevention. Diarylheptanoids (also known as diphenylheptanoids) are a small class of plant secondary metabolites that possess antioxidant activity greater than that of α-tocopherol. Curcumin is the best known member and is mainly extracted from turmeric. This study aimed to explore whether curcumin has a preventive effect on stroke.

**Methods:**

Stroke-prone spontaneously hypertensive rats (SHRsp) were randomly divided into control group (*n* = 10) and curcumin group (n = 10), and saline or curcumin (100 mg/kg/day) was administrated daily. Vascular endothelial function was examined by the relaxation of the artery in response to acetylcholine (ACH). The levels of reactive oxygen species (ROS) and nitric oxide (NO) were measured by using dihydroethidium (DHE) and 4, 5-diaminofluorescein (DAF-2 DA), respectively. The expression of uncoupling protein 2 (UCP2) was examined by RT-PCR and immunoblotting.

**Results:**

Administration of curcumin significantly delayed the onset of stroke and increased the survival of SHRsp, which was ascribed to decreased ROS and improved endothelial dependent relaxation of carotid arteries. In the presence of UCP2 inhibitor genipin, both curcumin-mediated decrease of ROS and increase of NO production were blocked.

**Conclusion:**

Our study suggests that curcumin exerts a stroke preventive effect by attenuating oxidative stress to improve vascular endothelial function, which might be associated with UCP2 signaling.

## Background

Stroke is an important cause of mortality and morbidity, accompanied with heavy economic and social burden worldwide. Although great advances have been made on the therapy of stroke, mortality and disability remain high. In addition, the absolute number of people affected by stroke worldwide increases significantly over years [1]. Therefore, prevention of stroke has been a major public health priority. Preventive strategies targeting high-risk individuals including diet and lifestyle changes have been proposed. Recently, mounting evidence showed that increase in the consumption of fruits is associated with reduced risk of stroke [2, 3]. However, the potential mechanism of dietary factors on stroke is not fully understood.

Among many dietary components, curcumin is a safe, natural polyphenol compound isolated from the plant *Curcuma longa*, which is a widely cultivated plant in the tropical regions of Asia. Curcumin is well recognized as a dietary spice for a long time and exerts a wide spectrum of biological functions including anti-cancer [4], cardioprotection [5], anti-inflammatory [6], antioxidant [7–9] and neuroprotection [10–12]. Notably, beneficial effects of curcumin on stroke have been suggested. Curcumin could decrease risk factors of stroke, such as lowering total cholesterol, boosting high density lipoprotein cholesterol level [13] and inhibiting platelet aggregation [14]. Thus, these properties make curcumin a potential drug for stroke prevention. Previous studies show that curcumin could attenuate ischemic stroke-induced brain injury due to its antioxidant activity [15]. It’s protective effect against stroke was also reported to be associated with its epigenetic modulatory properties [16], and neurogenesis by activating the Notch signaling pathway [17]. However, most of these studies focused on therapy, not prevention of stroke, whether or not curcumin prevents stroke occurrence is not know. Therefore, we hypothesize that curcumin plays a preventive effect on stroke. In the present study, SHRsp were used as the disease model due to its marked blood pressure elevation and predisposition in developing stroke. We showed the preventive effect of curcumin on stroke in SHRsp.

Vascular endothelial dysfunction plays critical role in pathogenesis of stroke. Vascular endothelial dysfunction, as reflected by impaired endothelium-dependent dilation (EDD), is associated with vascular oxidative stress [18]. Endothelium-derived NO, produced by the enzyme eNOS through oxidative conversion of L-arginine to L-citrulline, is important for EDD. It has been demonstrated that an increase in phosphorylation of eNOS delays the onset of stroke and increases the survival in SHRsp. Notably; curcumin was demonstrated to improve endothelial dysfunction and vascular remodeling by raising NO availability and reducing oxidative stress [19–21]. Our further study demonstrated the mechanisms underlying the protective effect of curcumin on stroke, and found that it is depend on UCP2 mediated anti-oxidant and NO production.

## Methods

All experiments adhered to the ARRIVE guidelines and were approved by the Third Military Medical University Animal Use and Care Committee.

### Animal

Male SHRsp, at 7 weeks of age, were obtained from the Shanghai Laboratory Animal Centre, Chinese Academy of Science. Rats were housed in the Animal Centre Care of Daping Hospital with a 12 h light/dark cycle and had ad libitum access to clean drinking water and rodent chow. After one week acclimation, the SHRsp were randomly divided into two groups: control group (saline) and curcumin group (100 mg/kg/day [22], gavage, Sigma-Aldrich, St. Louis, MO). Meantime, the SHRsp were fed with rodent chow containing 4% sodium chloride to induce stroke [23] until death. The blood pressure of the rats was measured by the tail-cuff method using a sphygmomanometer once every 2 weeks [24] (Model MLT 1030; Power Laboratory; AD Instruments, Sydney, Australia). For additional experiments after 4 weeks from the start of stroke-inducing, the rats were anesthetized with an intraperitoneal (IP) injection of pentobarbital sodium (60 mg/kg) and the carotid and basilar arteries were collected. The brain and blood samples were immediately harvested and stored at − 80 °C until use.

### Assessment of arteries relaxation in vitro

Arterial relaxation was measured using the carotid arteries of rats as previously described by Zhong MF et al. [25] Briefly, after the rats were anesthetized with an intraperitoneal injection of pentobarbital sodium (60 mg/kg body weight), the carotid arteries were carefully dissected and immediately placed in cold Krebs-Henseleit buffer (KHB) to remove the fat and connective tissues. They were then cut into ring segments with a length of 2–3 mm. One end of the ring was mounted on an organ bath with 95% O_2_ and 5% CO_2_ at 37 °C and the other end was connected to an isometric force transducer for tension measurement. After 60 min of equilibration under a resting tension of 0.5 g for carotid artery ring, the rings were incubated twice with KCl (60 mmol/L) to test for viability. The vasoconstriction or vasodilatation reaction to phenylephrine (PHE, 10^− 10^-10^− 5^ mmol/L, Sigma-Aldrich), acetylcholine (ACH, 10^− 9^-10^− 5^ mmol/L, Sigma-Aldrich) and sodium nitroprusside (SNP, 10^− 9^-10^− 5^ mmol/L) respectively were determined.

### Dyhidroethidium (DHE) staining

To assess superoxide production, the basilar artery was carefully and quickly isolated from SHRsp, as described previously by Zhu J et al. [26] After removal of connective tissues, the basilar artery was immediately frozen in embedding medium, cut into 8.0 μm thick sections and placed on chilled microscope slides. The samples were incubated in physiological saline solution (PSS) containing 10 μmol dihydroethidium (DHE; Sigma-Aldrich) for 30 min at 37 °C in the dark room. The vessels were then washed twice with PSS and placed under a fluorescence microscope (Nikon TE2000; Nikon Corporation, Tokyo, Japan). Images were obtained by using an excitation wave length of 520 to 540 nm and a rhodamine emission filter and the fluorescence intensity was analyzed. The level of superoxide in human umbilical vein endothelial cells (HUVECs) was analyzed similarly as described above.

### Measurement of NO level

NO levels in the vessels were assessed using DAF-2 DA (4, 5-diaminofluorescein, Sigma-Aldrich), as described by Donato et al. previously [6]. Arterial rings were incubated in 5 μmol/l DAF-2DA in the dark room for 30 min at 37 °C in PSS. Once the dye loading was completed, the vessels were rinsed three times with PSS. To measure the DAF fluorescence, the glass slides were placed under a Nikon E 600 fluorescence microscope (Nikon TE2000; Nikon Corporation, Tokyo, Japan) outfitted with a 40× PlanFluor objective and a fluorescein isothiocyanate filter set. The signals were acquired by using the software NIS-Elements 3.0 (Nikon), and the fluorescence intensity was assessed.

Accumulation of nitrate and nitrite in the blood was recognized as the end products of NO metabolism. The levels of nitrate and nitrite were measured as previously described in the method of Nakmareong S et al. [27] Briefly, plasma samples were deproteinized by ultrafiltration using centrifugal concentrators (Pall Corp., Ann Arbor, MI, USA). Then sample was mixed with 0.2 U/ml nitrate reductase and 1.2 μM NADPH, 4 mM glucose-6-phosphate disodium, 1.28 U/mL glucose-6 phosphate dehydrogenase. After being incubated for 30 min at 30 °C, the mixture was exposed to Griess solution (4% sulfanilamide in 0.3% napthylenediamine dihydrochloride) for 15 min. The light absorbance of the sample was measured on a spectrophotometer (Thermo Scientific) at 450 nm.

### Measurement of plasma malondialdehyde and superoxide dismutase

The concentration of plasma malondialdehyde (MDA) was calculated using the thiobarbituric acid (TBA) method, complying with the manufacturer’s protocol (Beyotime Institute of Biotechnology, Beijing, China). Plasma superoxide dismutase (SOD) activity was assayed using a Cu/Zn-SOD and Mn-SOD Assay Kit with WST-8 (Beyotime Institute of Biotechnology, Beijing, China) according to manufacturer’s protocol.

### RT-PCR

RT-PCR was conducted as previously described in the method of Huang H et al. [28] A total of 1.0 μg of RNA extracted from carotid artery tissues was used to synthesize cDNA that served as the template for amplification of the uncoupling protein 2 (UCP2). β-actin was used as an endogenous standard. For UCP2, the forward primer was 5’-ACCATTGCACGAGAGGAAGG-3′ and the reverse primer was 5′- TCTTGACCACATCAACGGGG -3′. For β-actin, the forward primer was 5’-GTGGGTATGGGTCAGAAGGA-3′ and the reverse primer was 5’-AGCGCG TAACCCTCATAGAT-3′. The amplification was performed under the following conditions: 35 cycles of denaturation for 2 min at 94 °C, annealing for 30 s at 62 °C, and extension for 45 s at 72 °C. Comparison of gene expression was performed using the ΔΔCT method.

### Cell culture and treatment

HUVECs were purchased from American Type Culture Collection (ATCC, Manassas, VA), cultured in Dulbecco’s modified eagle medium (DMEM) with 10% fetal bovine serum (FBS) in a humid atmosphere of 5% CO_2_ and 95% air at 37 °C. Cells between passage 3–5 were treated with H2O2 at different concentrations (10 μmol/L, 20 μmol/L, 50 μmol/L, 100 μmol/L) for 24 h with or without pretreatment with curcumin (0.01, 0.05, 0.1, 0.5, 1 and 5 μmol/L) for 2 h. Then cells were used for additional experiments.

### Cell viability

The Cell Counting Kit-8 (CCK-8) assay was performed to assess cell viability as previously described in the method of Han J et al. [29] In brief, cells were cultured in 96-well cell culture plate and pretreated with curcumin for 2 h and then treated with various concentrations of H2O2 for 24 h. Then after the medium was removed, 100 μl of fresh mixture (free serum DMEM 90 μl and 10 μl CCK-8 assay solution) was added to each well and incubated for an additional hour. The optical densities of the cells were assessed using a spectrophotometer (Thermo Scientific) at 450 nm. The cell viability was expressed as a ratio of optical density of treated versus control wells.

### Immunoblotting

Immunoblotting was performed as previously reported by Yang J et al. [30] Briefly, after boiling the homogenates in sample buffer (35 mmol/L Tris-HCl, pH 6.8, 4% SDS, 9.3% dithiothreitol, 0.01% bromophenol blue, 30% glycerol) at 95 °C for 5 min, 50 μg of protein were separated by SDS-PAGE (10% polyacrylamide), and then electroblotted onto nitrocellulose membranes (Bio-Rad). The blots were blocked overnight with 5% nonfat dry milk in PBST (0.05% Tween 20 in 10 mmol/l phosphate buffered (isotonic) saline) at 4 °C under constant shaking, then incubated with goat anti-UCP2 (Sigma-Aldrich, 1:250), rabbit anti-GAPDH (Sigma-Aldrich, 1:500) overnight in the cold-room at 4 °C. The membranes were then further incubated with infrared- labeled secondary antibodies (rabbit anti-goat or donkey anti-rabbit IRDye 800, Li-Cor Biosciences, Lincoln, NE) added to bind to the primary antibody at room temperature for 1 h. The membranes were washed three times with PBST. The bound complex was detected using the Odyssey Infrared Imaging System (Li-Cor Biosciences). The images were analyzed using the Odyssey Application Software to obtain the integrated intensities.

### Statistical analysis

The data are expressed as mean ± SEM. Comparison within groups were made by repeated measures analysis of variance with Duncan’s ad hoc test; comparison among groups(or t test when only two groups were compared) were made by factorial analysis of variance using Duncan’s ad hoc test. Stroke and survival curves were made using the Kaplan–Meier method and compared using the log-rank test. Significance was defined as a value of *P* < 0.05.

## Results

### General characteristics

**Fig. 1:**
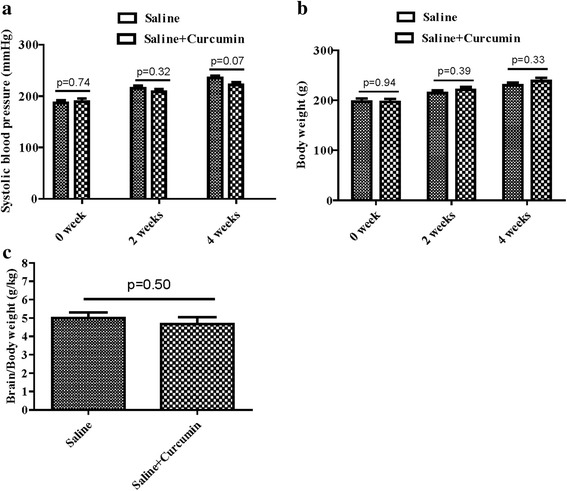
Characteristics of animals. System blood pressure (**a**), body weight (**b**), the ratio of brain (g)/body weight(kg) (**c**) were indicated from SHRsp with or without treatment by curcumin (100 mg/kg/day, *n* = 10)

To investigate the effect of curcumin on general characteristics of SHRsp, we measured blood pressure and body weight at 0, 2 and 4 weeks after curcumin treatment. Results showed that curcumin treatment did not influence blood pressure (*n* = 10 each group; Fig. 1a and Table 1), body weight (*n* = 10 each group; Fig. 1b and Table 1) and the ratio of brain weight (g)/body weight (kg) of SHRsp (n = 10 each group; Fig. 1c and Table 1).

**Table 1:** General characteristics of rats in saline group and saline + curcumin group

	Saline(*N* = 10)		Saline+Curcumin(N = 10)		*P* value
	Mean	SEM	Mean	SEM	
Systolic blood pressure (mmHg)					
0 week	186.30	5.71	189.10	6.13	0.74
2 weeks	215.50	4.56	208.00	5.77	0.32
4 weeks	235.30	4.57	221.50	5.64	0.07
Body weight (g)					
0 week	197.70	5.98	196.80	5.83	0.94
2 weeks	214.50	4.81	220.90	5.54	0.39
4 weeks	229.80	5.56	238.30	6.50	0.33
Brain/Body weight (g/kg)	5.01	0.29	4.69	0.37	0.50

### Curcumin treatment delayed the onset of stroke and increased survival time

**Fig. 2:**
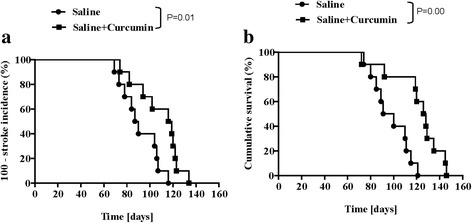
Effects of curcumin on incidence of stroke (**a**) and survival time (**b**) from SHRsp. The product limit (Kaplan-Meier) estimate of the cumulative stroke and survival was assessed with the log-rank test to evaluate for significant differences in stroke and survival. (n = 10)

To investigate whether curcumin treatment has a preventive effect on stroke, we observed the occurrence of stroke and death in all SHRsp with or without curcumin treatment. Compared to the saline group, curcumin significantly delayed the onset of stroke (n = 10 each group**;** Figs. 2a), which indicates preventive effect on stroke. Meanwhile, curcumin treatment led to a remarkable increase in survival time (121.20 ± 7.29 days versus 97.60 ± 5.09 days, n = 10 each group, *P* < 0.05; Figs. 2b), which may indicate protective effect on stroke.

### Curcumin treatment ameliorated arterial dysfunction in SHRsp

**Fig. 3:**
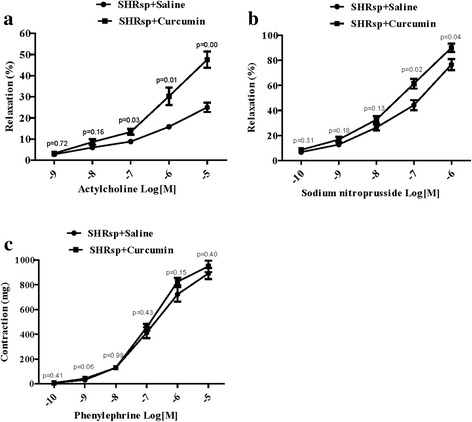
Effects of curcumin on carotid artery function in SHRsp. SHRsp was fed with curcumin (100 mg/kg/day,4 weeks); carotid artery from SHRsp was exposed to acetylcholine (ACH, *n* = 5 each group, **a**), sodium nitroprusside (SNP, n = 5 each group, **b**) and phenylephrine (PHE, *n* = 6 each group, **c**), the vasodilation or vasoconstriction was determined

Due to the role of the artery in the stroke, we checked arterial function in the carotid arteries of SHRsp. The results showed that the relaxation of carotid arteries response to ACH and SNP was in a dose-dependent manner. Compared with saline SHRsp, administration of curcumin significantly enhanced the relaxation of carotid artery response to ACH and SNP (*n* = 5 each group; Fig. 3a, b and Table 2), but did not change its response to PHE (*n* = 6 each group; Fig. 3c and Table 2).

**Table 2:** Comparison of arteries relaxation in vitro between saline group and saline + curcumin group

	Saline			Saline + Curcumin			P value
	Mean	SEM	N	Mean	SEM	N	
Relaxation (%) to actylcholine (Log[M])							
-9	2.80	0.66	5	3.20	0.86	5	0.72
-8	6.00	1.00	5	8.60	1.33	5	0.16
-7	8.80	1.07	5	13.40	1.44	5	0.03
-6	15.80	1.07	5	30.20	4.13	5	0.01
-5	25.00	2.17	5	47.60	3.83	5	0.00
Relaxation (%) to sodium nitroprusside (Log[M])							
-10	6.80	1.39	5	8.60	0.93	5	0.31
-9	12.80	1.56	5	16.80	2.22	5	0.18
-8	26.40	2.38	5	32.60	2.84	5	0.13
-7	44.20	4.09	5	61.40	3.93	5	0.02
-6	76.60	4.37	5	90.00	3.36	5	0.04
Contraction (mg) to phenylephrine (Log[M])							
-10	6.83	0.79	6	8.33	1.54	6	0.41
-9	30.83	2.71	6	42.17	4.54	6	0.06
-8	130.17	16.80	6	130.00	4.06	6	0.99
-7	413.50	43.84	6	456.17	28.35	6	0.43
-6	722.67	58.53	6	825.67	31.08	6	0.15
-5	891.67	45.46	6	948.83	46.27	6	0.40

### Curcumin treatment increased the NO levels, but decreased ROS production in basilar arterial wall and plasma from SHRsp

**Fig. 4:**
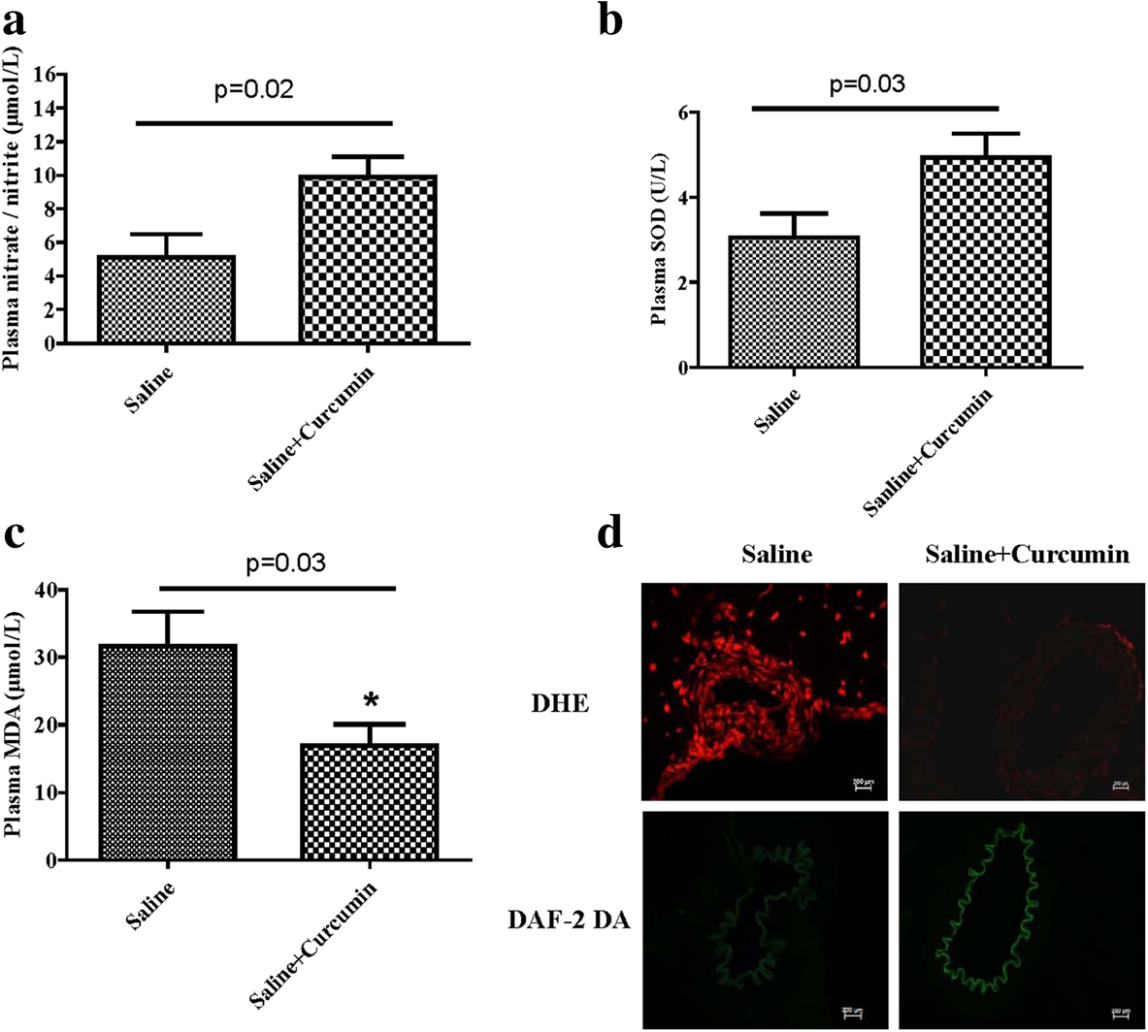
Effects of curcumin on NO and ROS accumulation in the basilar artery and plasma from SHRsp. SHRsp was fed with or without curcumin (100 mg/kg/day,4 weeks), the plasma levels of nitrate/nitrite (**a**), SOD (**b**) and MDA (**c**) were determined by assay kits (*n* = 8 each group). After the basilar arteries were isolated from SHRsp, NO and ROS expressions were determined by DAF-2 AF fluorescence (green, *n* = 3) and DHE staining (red, n = 3) (**d**)

Due to the role of ROS in the pathogenesis of stroke, we checked the ROS in the plasma and artery. The results showed that curcumin increased plasma nitrate/nitrite levels and plasma SOD activity (*n* = 8 each group; Fig. 4a, b and Table 3) and decreased plasma MDA levels (n = 8 each group; Fig. 4c and Table 3). The anti-oxidant effects were also investigated in the artery. We measured NO and ROS in the artery instead of SOD and MDA in the plasma, and found that curcumin increased NO levels, but decreased ROS levels in the basilar artery wall (*n* = 3 each group; Fig. 4d).

**Table 3:** Comparison of plasma nitrate/nitrite, SOD and MDA between saline group and saline + curcumin group

	Saline			Saline + Curcumin			P value
	Mean	SEM	N	Mean	SEM	N	
Plasma nitrate / nitrite (μmol/L)	5.11	1.38	8	9.91	1.20	8	0.02
Plasma SOD (U/L)	3.05	0.56	8	4.94	0.56	8	0.03
Plasma MDA (μmol/L)	31.69	5.06	8	17.01	3.05	8	0.03

### Role of UCP2 on the curcumin protection in artery

**Fig. 5:**
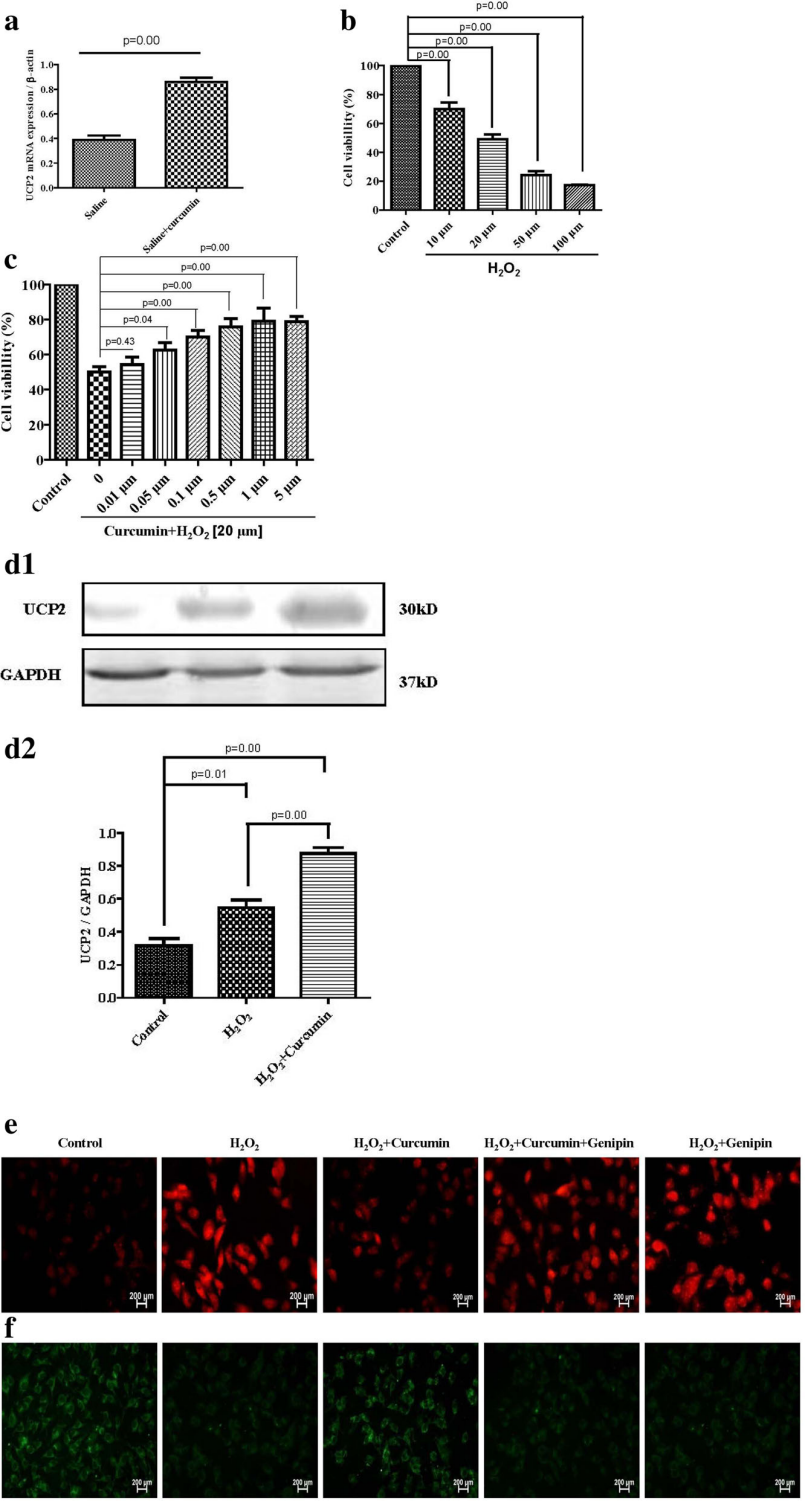
Role of UCP2 on the regulation of curcumin on ROS and NO accumulation in HUVECs_._ **a**: SHRsp was fed with curcumin (100 mg/kg/day) for 4 weeks, expression of UCP2 mRNA in carotid artery SHRsp was determined by RT-PCR, Result was indicated as ratio of UCP2 and β-actin. (n = 6). **b**: HUVECs were exposed to different doses of H_2_O_2_ (10, 20, 50, 100 μmol) for 24 h. Cell viability was measured by CCK-8 assay (*n* = 7). **c:** HUVECs were pre-treated with curcumin (0.01, 0.05, 0.1, 0.5,1, 5 μmol/L) for 2 h and then co-incubated with H_2_O_2_ (20 μmol/L) for 24 h. The cell viability was measured by CCK-8 assay (n = 7). **d-f**: HUVECs were exposed to different reagents (H_2_O_2_ 20 μmol/L, curcumin 1 μmol/L, genipin 10 μmol/L). UCP2 expression was examined by western blot (**d**). Representative images of ROS (**e**) and NO (**f**) staining were shown (D: n = 5; E and F were repeated at least three times)

UCP2 is a member of the mitochondrial anion carrier family and a physiological regulator of mitochondrial ROS generation [31]. To check whether UCP2 is involved in the anti-oxidative effect of curcumin, UCP2 expression was determined by RT-PCR. Results showed that curcumin significantly increased UCP2 mRNA levels in the carotid artery from SHRsp (*n* = 6 each group; Fig. 5a).

The effect of curcumin on UCP2 was also confirmed in the HUVECs using H_2_O_2_ to simulate oxidative stress in vitro. Results showed that H_2_O_2_ significantly decreased cell viability, which was attenuated by curcumin in a concentration-dependent manner (*n* = 7 each group; Fig. 5b and c). Moreover, the UCP2 expression was promoted by curcumin (*n* = 5 each group; Fig. 5d). Consistent with the in-vivo study, curcumin increased NO levels, decreased ROS levels in the HUVECs (n = 3 each group; Fig. 5e and f). However, in the presence of UCP2 inhibitor, genipin (10 μmol/L), the effects of curcumin on ROS and NO were blocked (Fig. 5e and f), indicating that UCP2 is an important signaling factor for the antioxidant role of curcumin.

## Discussion

In the current study, we demonstrated that curcumin exerts a preventive effect on stroke supported by a latency phenotype of SHRsp. This effect might be associated with improvement of vascular endothelial function via UCP2 mediated anti-oxidant effect.

Previous studies showed that consumption of fruits and vegetables, including apple, pear, and leafy vegetables, lowers the risk of stroke [2, 32, 33]. For example, Sparassis crispa, an edibile mushroom, has been shown to have a preventive effect for stroke through amelioration cerebrovascular endothelial dysfunction [34]. Our present study demonstrated that treatment with curcumin significantly delayed the onset of stroke and markedly increased the survival time of SHRsp, supporting that diet change may be an effective way to prevent stroke and suggesting turmeric as a good choice for patients at high risk of stroke. In this study, we did not observed the effect on blood pressure, indicating the protective effect is independent of lowering blood pressure in SHRsp.

Ameliorating the endothelial dysfunction has showed a preventive effect in SHRsp [24, 34]. It is well known that endothelial NO is an endogenous mediator of vasodilatation. In addition, oxidative stress also plays an important role in stroke. The decreased NO production or availability, at least in part, is due to excess superoxide anion in SHRsp [35]. Therefore, enhancement of NO level and reduction of oxidative stress might be feasible strategies to prevent stroke occurrence. Curcumin has shown its beneficial effect in improvement of endothelial function through raising nitric oxide availability and reducing oxidative stress. Our present study found that curcumin increased NO levels, decreased ROS levels in plasma and basilar artery and significantly improved endothelial dysfunction in SHRsp. This protective effect against endothelial dysfunction was consistent with previous studies [19, 21]. Notably, SNP leads to release of NO thus dilating artery and in our study EDD with SNP was promoted by curcumin. Therefore, we speculate that SNP and curcumin have synergistic effect in increasing NO levels thus combined administration might be beneficial for preventing stroke.

UCP2 is a member of the mitochondrial uncoupling proteins, belonging to the mitochondrial inner anion carrier family, which mediates the proton leak and control of superoxide [31]. Evidence suggests that UCP2 is a negative regulator of ROS generation within the mitochondrion [36]. It has been demonstrated that UCP2 inhibition leads to a significant increase of oxidative stress in endothelial cells, whereas overexpression of UCP2 inhibits endothelial cells apoptosis and ameliorates vascular endothelial function by inhibiting ROS production [37–39]. In addition, upregulation of UCP2 expression plays an important role in the restoration of hyperglycemia-induced endothelial dysfunction, which is due to the suppression of superoxide production and the elevation of NO production in the blood vessels and endothelial cells [40]. Therefore, we examined whether UCP2 involved in the endothelial protection of curcumin. In the presence of UCP2 blocker, the effects of curcumin on ROS and NO productions were blocked. Thus we speculate that UCP2 signaling is involved in the preventive effect of curcumin on stroke in hypertension via the decreasing ROS and increasing NO levels.

Mitochondria is suggested by many studies as a target in the therapeutic properties of curcumin in many disease [41]. In cancer state, curcumin was observed to induce permeability transition pore opening and production of ROS, thus inducing apoptosis of cancer cells and exerting anti-cancer effect [42]. On the contrary, in physiological conditions, it was observed to induce cytoprotective enzymes and ROS diminishing, thus exerting anti-oxidant effect [43]. The mechanism by which curcumin exerts its dual role in normal and cancer cells is still unknown and remains to be explored. Our study showed curcumin decreases ROS in artery and HUVECs, and this is helpful to understand the protective effect of curcumin on stroke. It should be noticed that the detailed mechanisms of curcumin on ROS production need to be investigated in the future.

## Conclusion

Despite increased knowledge in controlling risk factors of stroke, how to control stroke remains challenging. Curcumin has been shown to have vascular protective benefits in the arteries. In our present study, we report that administration of curcumin significantly delayed the onset of stroke and increased the survival time for SHRsp. Interestingly, this effect is independent of a reduction in blood pressure. Instead, reduction of oxidative stress, via a UCP2/NO pathway, is involved in the preventive process.

## Abbreviations

ACH: Acetylcholine
CCK-8: Cell Counting Kit-8
DAF-2 DA: 4, 5-diaminofluorescein
DHE: Dihydroethidium
DMEM: Dulbecco’s Modified Eagle Medium
EDD: Endothelium-dependent Dilation
FBS: Fetal Bovine Serum
HUVECs: Human Umbilical Vein Endothelial Cells
IP: Intraperitoneal
KHB: Krebs-Henseleit Buffer
MDA: Malondialdehyde
NO: Nitric Oxide
PHE: Phenylephrine
PSS: Physiological Saline Solution
ROS: Reactive Oxygen Species
SHRsp: Stroke-prone Spontaneously Hypertensive Rats
SNP: Sodium Nitroprusside
SOD: Superoxide Dismutase
TBA: Thiobarbituric Acid
UCP2: Uncoupling Protein 2
